# Microstructure and Mechanical Properties of Spark Plasma Sintered Si_3_N_4_/WC Ceramic Tools

**DOI:** 10.3390/ma12111868

**Published:** 2019-06-10

**Authors:** Zhenhua Wang, Jiheng Jia, Liyan Cao, Ning Sun, Yulin Wang

**Affiliations:** School of Mechanical Engineering, Nanjing University of Science and Technology, Nanjing 210094, China; jihengjia1993@163.com (J.J.); liyan_cao@126.com (L.C.); sunningbos@163.com (N.S.)

**Keywords:** silicon nitride, tungsten carbide, mechanical properties, spark plasma sintered

## Abstract

Silicon nitride (Si_3_N_4_) based ceramic tools exhibit good machinability in cutting materials such as gray cast iron, ductile iron, malleable cast iron, and superalloys due to excellent high-temperature mechanical properties. In this paper, high-performance Si_3_N_4_-based ceramic tools containing tungsten carbide (WC) and cobalt (Co) were studied. Effects of the WC content and Co content on mechanical properties and a microstructure of Si_3_N_4_-based ceramic materials were analyzed. Results showed that Si_3_N_4_-based ceramic material containing 10 wt % WC and 1 wt % Co had the best comprehensive mechanical properties at a sintering temperature of 1650 °C and holding time of 6 min, achieving Vickers hardness, fracture toughness, and room temperature bending strength of 16.96 GPa, 7.26 MPa·m^1/2^, and 1132 MPa, respectively. The microstructure of Si_3_N_4_-based ceramic tool material is uniform without obvious abnormal growth. The Si_3_N_4_-based ceramic tool was mainly composed of α-Si_3_N_4_, β-Si_3_N_4_, and WC phases.

## 1. Introduction

Silicon nitride ceramic materials are widely used in much of the industrial equipment such as cutting tools, gas turbines, thermal insulation materials, and engine parts due to their high strength, high elastic modulus, wear resistance, thermal shock resistance, and creep resistance [1,2,3]. Silicon nitride ceramic cutting tools have good cutting performance in processing the gray cast iron, nodular cast iron, malleable cast iron, and high-temperature alloy. At present, the main factors affecting the cutting performance of silicon nitride ceramic tools are low hardness and fracture toughness. With the aim to improve the hardness and fracture toughness of Si_3_N_4_-based ceramic material, scholars had made many efforts such as adding a hard phase, nano-phase, binder phase, and lubricant phase to Si_3_N_4_-based ceramic tool materials [4,5].

Adding a high hardness second phase is an important means to improve the hardness and fracture toughness of silicon nitride ceramics. The second phase, which is commonly added to the Si_3_N_4_-based composite materials, mainly includes the titanium diboride (TiB_2_) [6], titanium carbide (TiC) [7,8], titanium nitride (TiN) [9], Ti (C_7_N_3_) [10], silicon carbide (SiC) [11], carbon nanostructure [12], and (W, Ti)C [13], and the like. The choice of the second phase is particularly important because it has a great impact on the microstructure and properties of a Si_3_N_4_-based composite. Tapasztó et al. [14] prepared the Si_3_N_4_-based nanocomposites by hot isostatic pressing sintering (HIP) and spark plasma sintering (SPS) using single- and multi-walled carbon nanotubes (SWCNT and MWCNT), exfoliated graphite (GR), and carbon black (CB) as the reinforcing phases. Their results showed that the comprehensive mechanical properties of the Si_3_N_4_-based nanocomposites prepared by the two sintering methods were worse than those of the single-phase silicon nitride ceramics without carbon nanostructures. The Si_3_N_4_-based nanocomposites prepared by the hot isostatic pressing, which had good toughness, was mainly composed of the β-Si_3_N_4_. The sample prepared by the spark plasma sintering, which had high hardness, was also mainly composed of α-Si_3_N_4_. Xu et al. [13] prepared the Si_3_N_4_/(W, Ti)C composite ceramic tool materials by microwave sintering, and found that (W, Ti)C inhibited the densification and phase transformation of the Si_3_N_4_-based composite ceramics. Besides, they found that when the sintering temperature decreased by 100 °C, the mechanical properties of the Si_3_N_4_-based matrix composites increased by 6–9%.

At present, the methods for preparation of Si_3_N_4_-based ceramic material are mainly based on the traditional sintering methods such as hot pressing [14,15], hot isostatic pressing [12], air pressure [16], pressureless [17], and reaction sintering [18,19]. In addition, there are new preparation techniques such as spark plasma sintering [20,21,22] and microwave sintering [3,13]. The traditional sintering methods have the disadvantages of high energy consumption and low efficiency, and the microwave sintering is unevenly heated, so a sample is easy to crack, which seriously affects the performance reliability and economic feasibility of the tool. The SPS denotes a new type of material preparation methods which provides high efficiency, has simple operation, low energy consumption, and environmental protection. The SPS technology has the characteristics of hot pressing, plasma activation, and current heating, which make the material obtain high density, uniform microstructure, and fine grains [23]. Therefore, SPS technology was chose to sinter Si_3_N_4_-based ceramic.

In addition, the mechanical properties of Si_3_N_4_-based ceramic can be significantly improved by appropriate sintering additives and composition ratio. Common sintering aids are oxides, nitrides, and fluorides. These sintering aids play different roles in the preparation of Si_3_N_4_-based ceramic. MgO is more significant in reducing the sintering temperature [3]. CeO_2_ has better wettability with silicon nitride [3]. MgSiN_2_ can increase the density of Si_3_N_4_ ceramic [21]. In our previous study, MgSiN_2_-Y_2_O_3_-CeO_2_ system was selected as sintering aids to analyze the effects of various components on the mechanical properties and microstructures of Si_3_N_4_-based ceramic materials. The results shown that Si_3_N_4_-based ceramic materials with 5 wt % MgSiN_2_, 3 wt % Y_2_O_3_, and 1 wt % CeO_2_ sintering aids had the best mechanical properties [22]. Therefore, the sintering aids of 5 wt % MgSiN_2_, 3 wt % Y_2_O_3_, and 1 wt % CeO_2_ were selected in this paper.

WC has the advantages of high fracture toughness, good wear resistance, high hardness, and good chemical stability. In addition, in the spark plasma sintering process of ceramic materials, adding an appropriate amount of conductive metal phase (such as Co, Ni, Mo, etc.) can increase the current through the Si_3_N_4_-based ceramic material, promote Joule heat generation and increase the α→β Si_3_N_4_ phase transformation rate. The wettability between WC and metal Co is good. The WC-Co cemented carbide prepared by spark plasma sintering technology not only has a small grain size, but also greatly improves the mechanical properties [24]. Therefore, WC and Co are selected as the phase to improve the properties of the Si_3_N_4_ ceramic tool. Effects of the WC and Co content on the mechanical properties and microstructure of Si_3_N_4_-based ceramic material are investigated in the paper.

In this paper, Si_3_N_4_-based ceramic material was prepared by adding WC and Co by SPS. The effects of WC and Co content on relative density, microstructure, and mechanical properties of Si_3_N_4_/WC ceramic material were investigated. The bending strength of the Si_3_N_4_/WC ceramic materials at different temperatures were analyzed to obtain a Si_3_N_4_-based ceramic material with excellent comprehensive mechanical properties and to provide support for machining hard materials.

## 2. Experimental Methods

### 2.1. Sample Preparation

The raw powders (purity: 99.99%) were purchased from Shanghai Chaoyu New Material Technology Co. Ltd (Shanghai, China). The masses of the α-Si_3_N_4_ (700 nm), WC (500 nm), Co (500 nm), MgSiN_2_ (500 nm), Y_2_O_3_ (1 μm), and CeO_2_ (1 μm) powders were weighed on an electronic balance (WH-BL2003, Weiheng, Guangzhou, China) in proportion, which were then placed in a polyurethane ball mill tank and ball milled in a planetary ball mill (QM-3SP2, Nanda, Nanjing, China) with silicon nitride balls for 48 h. The ratio of powder to ball was 7:1. The mixed powder after ball milling was dried at 120 °C in a vacuum drying oven (DZF-1 type, Keheng, Shanghai, China), and then ground and passed through a 100-mesh sieve. Twenty grams of mixed powder was loaded into a graphite mold having an inner diameter of 20 mm, and a graphite paper coated with boron nitride was placed between the mold and powder for the purpose of more easily demolding after sintering. Subsequently, a graphite mold wrapped with carbon felt was placed in a chamber of a spark plasma sintering system (LABOXTM-650F, Sinter Land Inc., Nagaoka, Japan) for sintering (1650 °C sintering temperature, 100 °C/min heating rate, 50 MPa sintering pressure, and 6 min holding time at Ar). After the sintered body was coarsely grinded, grinded, and polished, the properties were analyzed and characterized.

The experiments with two factors and three levels were conducted at a sintering temperature of 1650 °C while holding for 6 min. The WC content was 5 wt %, 10 wt %, and 15 wt %, and the Co content was 1 wt %, 2 wt %, and 4 wt %, respectively. The total content of the sintering aid was 9 wt % (the specific composition consisted of 5 wt % MgSiN_2_, 3 wt % Y_2_O_3_, and 1 wt % CeO_2_).

### 2.2. Performance Testing

The sample density was determined by the Archimedes’ drainage method, and the relative sample density was calculated using the ratio of the measured bulk density to the theoretical density. The Vickers hardness and the fracture toughness of a sample were determined by the indentation method. The load was 196.1 N, and it was maintained for 15 s. The Vickers hardness and fracture toughness were respectively defined by [25]:

(1) $$H_{V}=0.001854\times \frac{F}{d^{2}},$$

(2) $$K_{IC}=0.203{\left(c/a\right)}^{-3/2}\sqrt{a}H_{V},$$

where *H_V_* denoted the Vickers hardness of a sample (given in GPa), *F* denoted the load (given in N), *d* represented the diagonal length of the material indentation (given in mm), *K_IC_* was the fracture toughness (given in MPa·m^1/2^), *c* denoted a half of the crack length (given in mm), and lastly, *a* denoted the half-length of the diagonal indentation (given in mm).

Bending strength of the Si_3_N_4_-based ceramic material containing 10 wt % WC and 1 wt % Co (named the SW10) at different temperatures (20, 200, 400, 600, and 800 °C) was measured by the method of a 3-points bending test using an electronic universal testing machine (UTM5105-G, Hengsisheng, Jinan, China). The size of bending strength-testing sample was 30 mm × 5 mm × 5 mm (length × width × height). The phase composition of the samples was analyzed by a Bruker D8 X-ray diffractometer (XRD, D8 Advance, Bruker, Leipzig, Germany). The microstructure and elemental composition were examined with a scanning electron microscope (SEM, Quant250FEG, FEI, Hillsboro, OR, USA) equipped with an energy dispersive spectrometer (EDS).

## 3. Results and Discussion

**Figure 1:**
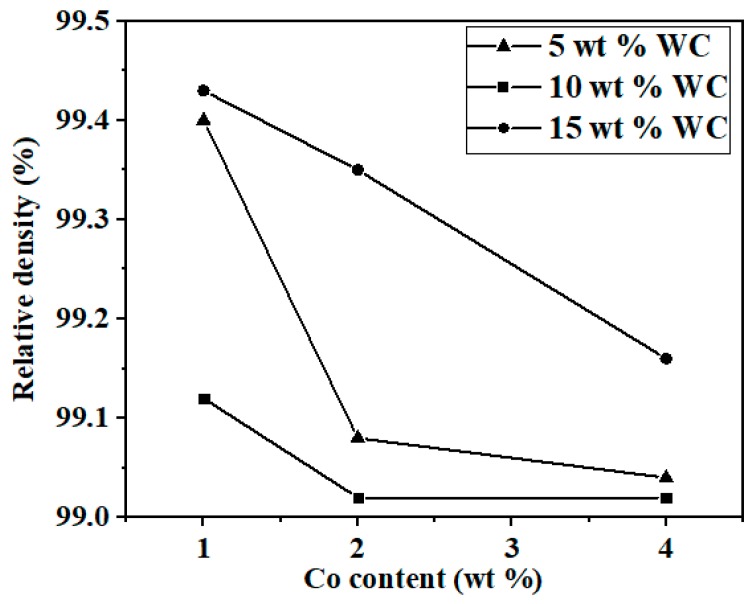
Relative density of the Si_3_N_4_/WC ceramic tool material at different WC and Co contents.

The relationship between the relative density of Si_3_N_4_-based ceramic materials and the WC and Co content was presented in Figure 1, wherein it can be seen that the density of all the sintered samples was above 99%. Generally, the spark plasma sintered Si_3_N_4_-based ceramic material could obtain higher density.

**Figure 2:**
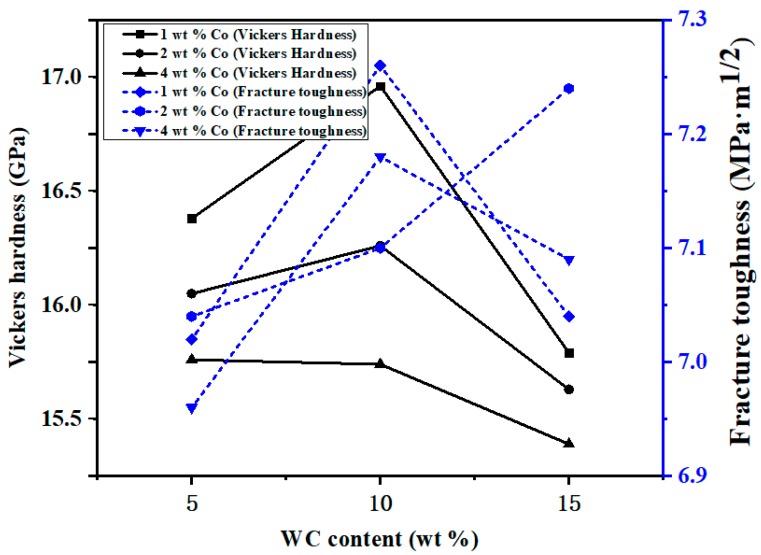
Vickers hardness and fracture toughness of the Si_3_N_4_/WC ceramic tool at different Co content and WC content.

Effects WC and Co content on the hardness and fracture toughness of the Si_3_N_4_-based ceramic material is presented in Figure 2. As shown in Figure 2, the Vickers hardness of the sample firstly increased, and then decreased with the increase in WC content. The Vickers hardness of the sample decreased with the increase in Co content. The fracture toughness firstly increased and then decreased with the increase in WC content when the Co content was 1 wt % and 4 wt %. However, the fracture toughness increased with the increase in WC content when the Co content was 2 wt %. In summary, the Si_3_N_4_-based ceramic material containing 10 wt % WC and 1 wt % Co (named the SW10) achieved the best comprehensive mechanical properties, and the Vickers hardness, fracture toughness were 16.96 GPa and 7.26 MPa·m^1/2^, respectively. Compared with Si_3_N_4_-based ceramic material (91 wt % Si_3_N_4_ + 5 wt % MgSiN_2_ + 3 wt % Y_2_O_3_ + 1 wt % CeO_2_) [22], the Vickers hardness and fracture toughness of SW10 ceramic material were increased by 2.60% and 5.37%, respectively.

**Figure 3:**
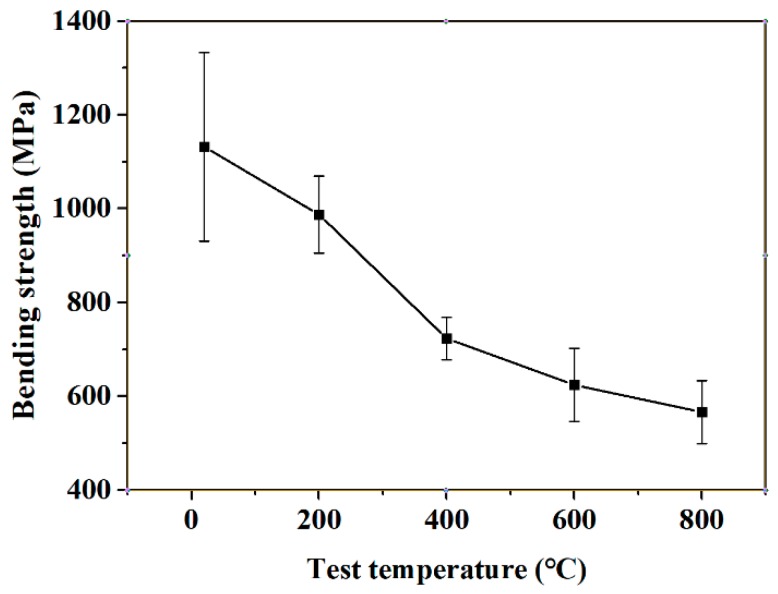
Bending strength of the Si3N4-based ceramic material containing 10 wt % WC and 1 wt % Co at different temperatures.

SW10 ceramic tools must bear higher cutting temperature during cutting processes. The cutting performance of the cutting tool is better when the cutting tool material has high mechanical properties at a high temperature. In order to study the effect of temperature on the bending strength of SW10, the bending strength of SW10 ceramic materials were analyzed when the material temperature was 20, 200, 400, 600, and 800 °C. The bending strength of the SW10 ceramic material at different temperatures were presented in Figure 3. As shown in Figure 3, the bending strength of SW10 ceramic material reached 1132 MPa at room temperature of 20 °C, and the bending strength reached the value of 566 MPa when the temperature reached 800 °C, which demonstrated excellent high-temperature mechanical properties. The bending strength of the material generally decreased with the increase of temperature.

**Figure 4:**
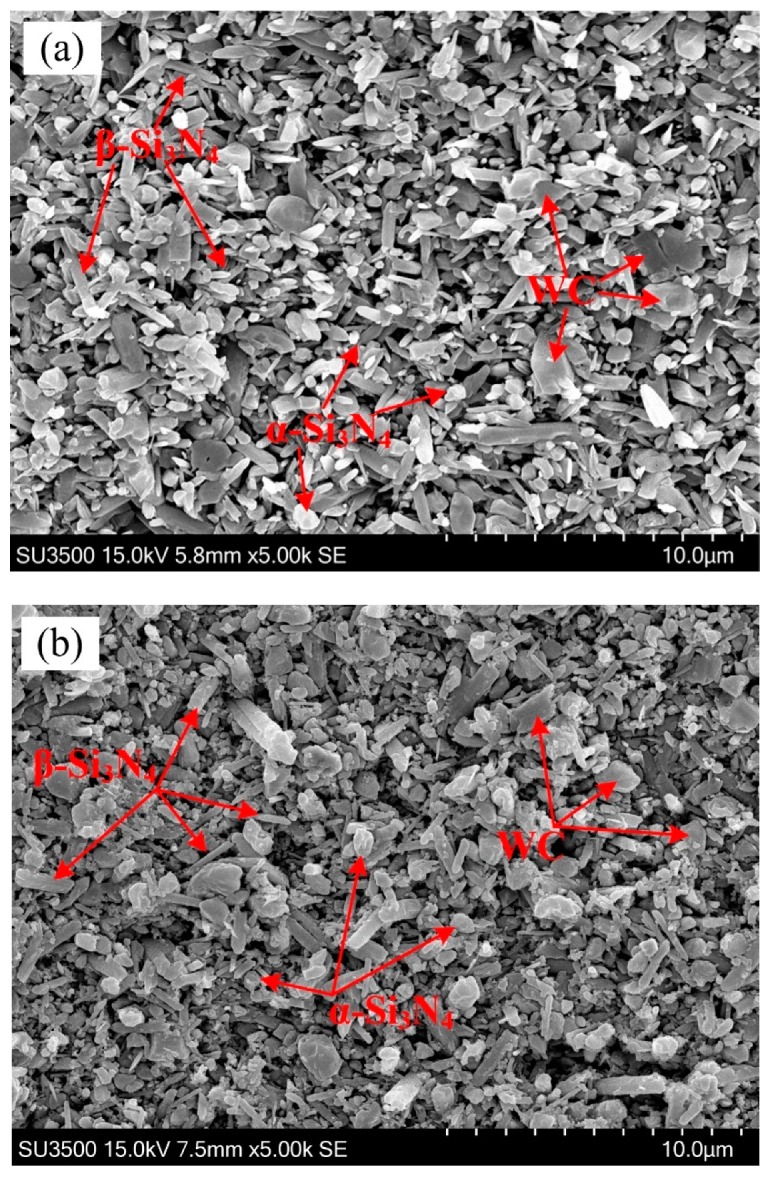

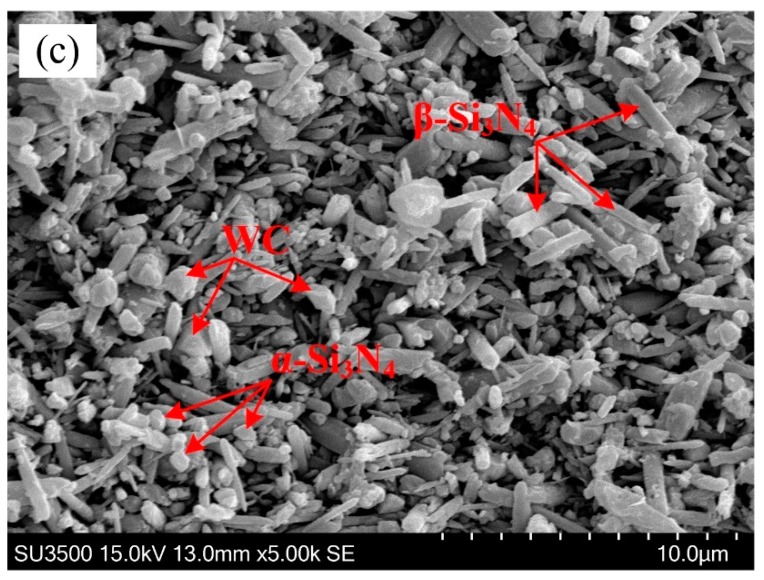
SEM micrograph of the Si_3_N_4_-based ceramic with 1 wt % Co after being corroded. (**a**) 5 wt % WC, (**b**) 10 wt % WC, and (**c**) 15 wt % WC.

In Figure 4, the SEM micrographs of the Si_3_N_4_-based ceramic having different WC content with the Co content of 1 wt % after being corroded by the molten NaOH were respectively presented. As shown in Figure 4, no obvious abnormal growth was observed in the Si_3_N_4_ grains under different conditions; the microstructure was relatively uniform and small, and the aspect ratio of the β-Si_3_N_4_ phase was high. Sintering of the Si_3_N_4_-based ceramic materials was a dissolution and reprecipitation process [26,27]. The sintering aid formed a low eutectic liquid phase with the SiO_2_ oxide film on the surface of the silicon nitride ceramic at high temperature. When the temperature exceeded 1500 °C, the α-Si_3_N_4_ phase began to convert into β-Si_3_N_4_ phase silicon nitride according to the Ostwald ripening mechanism. First, smaller α-Si_3_N_4_ grains first dissolved into the liquid phase, and when the liquid phase was saturated, then larger β-Si_3_N_4_ grains precipitated. Since the growth rate of the β-Si_3_N_4_ grains in different directions was very different, the β-Si_3_N_4_ grains tended to grow in preferential orientation to form the rod-like grains [28]. The length-diameter ratio of β-Si_3_N_4_ grain was calculated by measuring the lengths and diameters of 50 randomly selected β-Si_3_N_4_ grains. Statistical results shown that at the WC content of 5 wt %, 10 wt %, and 15 wt %, the average value of length-diameter ratio of β-Si_3_N_4_ was 4.45, 7.81, and 3.26, respectively. Therefore, the average value of length-diameter ratio of β-Si_3_N_4_ was largest when the WC content was 10 wt %, which leaded to higher fracture toughness of Si_3_N_4_-based ceramic materials containing having 10 wt % WC.

**Figure 5:**
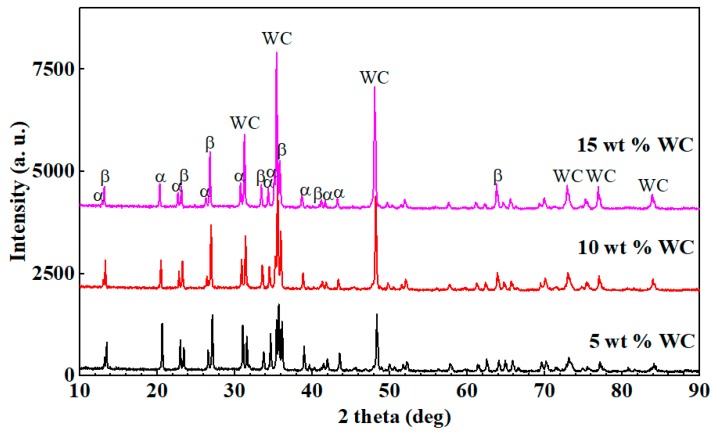
Effect of the WC content on the phase composition of the Si_3_N_4_-based ceramic material.

The X-ray diffraction patterns of the Si_3_N_4_-based ceramic having different WC contents at the Co content of 1 wt % are presented in Figure 5. The quantitative analysis of α-Si_3_N_4_ and β-Si_3_N_4_ of the samples was performed using the diffraction intensities of the (1 0 1), (2 0 1), (1 0 2), and (3 0 1) planes for α-Si_3_N_4_, and of the (1 1 0), (2 0 0), (1 0 1), and (2 1 0) planes for β-Si_3_N_4_ [29]. As Figure 4a and Figure 5 show, the α-Si_3_N_4_ grains constituted the majority in the Si_3_N_4_-based ceramic material having 5 wt % WC, while there were fewer β-Si_3_N_4_ grains and the α→β-Si_3_N_4_ conversion rate was 37.91%, As shown in Figure 4b and Figure 5, the number of β-Si_3_N_4_ grains in the Si_3_N_4_-based ceramic with the WC content of 10 wt % was remarkably increased compared with the Si_3_N_4_-based ceramic material having 5 wt % WC, and the α→β-Si_3_N_4_ conversion rate was 46.21%. The formation of long columnar grains had an important influence on the fracture toughness of the Si_3_N_4_-based ceramic material. As shown in Figure 4b, many well-developed long columnar grains were stacked together forming a spatial network structure. The spindly β-Si_3_N_4_ grains were staggered with each other, preventing the crack expansion, and improving the fracture toughness of the material. The columnar β-Si_3_N_4_ increased gradually when the WC content increased to 15 wt %, and the conversion rate of α→β-Si_3_N_4_ was 47.27%, as shown in Figure 4c and Figure 5. In the non-porous region, some short rod-like β-Si_3_N_4_ grains were embedded in the network structure. As shown in Figure 4b,c the β-Si_3_N_4_ grain size of the Si_3_N_4_-based ceramic material containing 15 wt % WC was larger than the β-Si_3_N_4_ grain size of the Si_3_N_4_-based ceramic material containing 10 wt % WC. When the WC content was 15%, the porosities were easily formed between the coarse grains of β-Si_3_N_4_, which led to a decrease in fracture toughness (as shown in Figure 2). Therefore, higher Vickers hardness and fracture toughness can be obtained at a WC content of 10 wt %.

**Figure 6:**
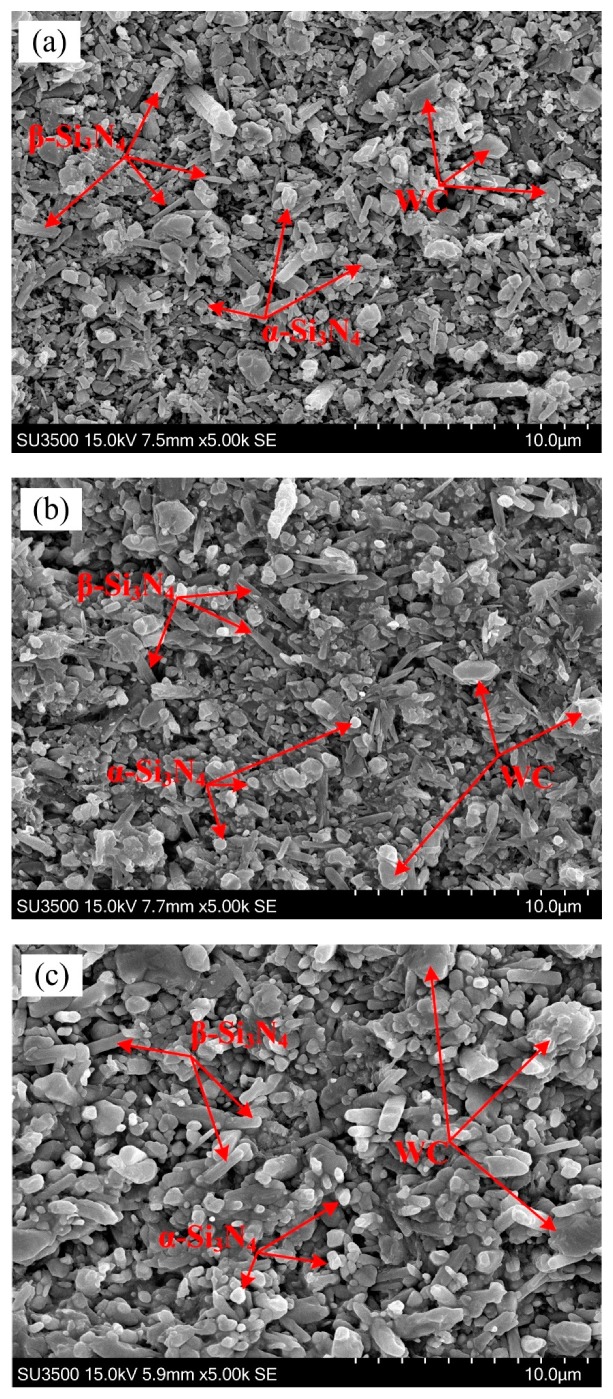
SEM micrograph of the Si_3_N_4_-based ceramic with 10 wt % WC after being corroded. (**a**) 1 wt % Co, (**b**) 2 wt % Co, and (**c**) 4 wt % Co.

**Figure 7:**
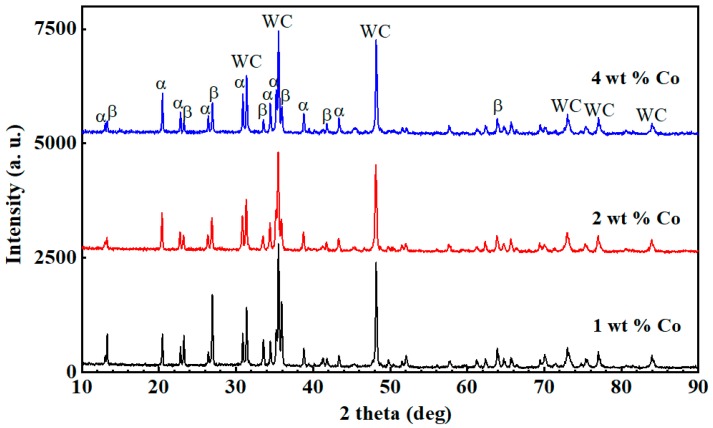
Effect of the Co content on the phase composition of Si_3_N_4_-based ceramic material at 10 wt % WC.

The SEM micrographs and X-ray diffraction pattern of the corroded surface of Si_3_N_4_-based ceramic having different Co content with the WC content of 10 wt % were respectively presented in Figure 6 and Figure 7. As shown in Figure 6 and Figure 7, the amount and length-diameter ratio of β-Si_3_N_4_ decreased with the increase of cobalt content, and some larger WC grains were formed. Statistical results shown that at the Co content of 1 wt %, 2 wt %, and 4 wt %, the average value of length-diameter ratio of β-Si_3_N_4_ was 7.81, 4.62, and 5.83, respectively.

As Figure 7 showing, the intensity of the β-Si_3_N_4_ phase diffraction peak was decreased with the increase in Co content, and the intensity of the α-Si_3_N_4_ phase diffraction peak was slightly increased with the increase in Co content. When the Co content is 1 wt %, 2 wt %, and 4 wt %, the conversion rate of α→β-Si_3_N_4_ was 46.21%, 35.71%, and 30.28%, respectively. The results showed that the conversion rate of α→β-Si_3_N_4_ decreased with the increase of Co content. This was mainly because the WC and Co had good wettability, and when the WC powder was sintered by the spark plasma sintering method under the action of the Co powder, the densification sintering process could be completed at the sintering temperature of 1250 °C [28], and such a temperature was significantly lower than the high densification sintering temperature (1650 °C) of α-Si_3_N_4_ under the action of the sintering aid. When the Co content increased, the WC and Co powders in the Si_3_N_4_-based ceramic material had more chances to contact with each other, providing more sintering power for the WC pre-sintering. Therefore, the WC grains formed in the initial stage of sintering were uniformly distributed in the Si_3_N_4_-based ceramic material, which hindered the conversion of α-Si_3_N_4_ to β-Si_3_N_4_. Meanwhile, those WC grains might also hinder the overall columnar growth of β-Si_3_N_4_. So, higher Vickers hardness and fracture toughness can be obtained at 1 wt % Co when WC content was 10 wt %.

## 4. Conclusions

In this paper, the high-performance Si_3_N_4_-based ceramic material tools containing the WC and Co are prepared by spark plasma sintering to analyze the effect of the WC content and Co content on the phase composition, the conversion rate of α→β-Si_3_N_4_, hardness, fracture toughness, and microstructure of the Si_3_N_4_-based ceramic material tools. The main conclusions are as follows.

(1) At the sintering temperature of 1650 °C the Si_3_N_4_-based ceramic material containing 10 wt % WC and 1 wt % Co (SW10) achieved the best Vickers hardness and fracture toughness, and the Vickers hardness, fracture toughness, and room temperature bending strength were 16.96 GPa, 7.26 MPa·m^1/2^, and 1132 MPa respectively, and the α→β phase conversion rate was 46.21%.
(2) The microstructure image of the Si_3_N_4_-based ceramic material showed that there were no abnormally grown grains at the different WC content and Co content, and the microstructure was uniform. The content of β-Si_3_N_4_ phase increased with the increase in WC content at the Co content of 1 wt %, but the content of β-Si_3_N_4_ phase decreased with the increase in Co content at the WC content of 10 wt %.
